# Prediction of hydrogenated group IV–V hexagonal binary monolayers

**DOI:** 10.1038/s41598-020-71766-5

**Published:** 2020-09-11

**Authors:** Mohammad Ali Mohebpour, Shobair Mohammadi Mozvashi, Sahar Izadi Vishkayi, Meysam Bagheri Tagani

**Affiliations:** 1 Computational Nanophysics Laboratory (CNL), Department of Physics, University of Guilan, P. O. Box 41335-1914, Rasht, Iran; 2 School of Physics, Institute for Research in Fundamental Sciences (IPM), P. O. Box 19395-5531, Tehran, Iran

**Keywords:** Condensed-matter physics, Materials for devices, Materials for optics, Nanoscale materials

## Abstract

Group IV and V monolayers are very crucial 2D materials for their high carrier mobilities, tunable band gaps, and optical linear dichroism. Very recently, a novel group IV–V binary compound, ${\hbox {Sn}}_2{\hbox {Bi}}$, has been synthesized on silicon substrate, and has shown very interesting electronic properties. Further investigations have revealed that the monolayer would be stable in freestanding form by hydrogenation. Inspired by this, by means of first-principles calculations, we systematically predict and investigate eight counterparts of ${\hbox {Sn}}_2{\hbox {Bi}}$, namely ${\hbox {Si}}_2{\hbox {P}}$, ${\hbox {Si}}_2{\hbox {As}}$, ${\hbox {Si}}_2{\hbox {Sb}}$, ${\hbox {Si}}_2{\hbox {Bi}}$, ${\hbox {Ge}}_2{\hbox {P}}$, ${\hbox {Ge}}_2{\hbox {As}}$, ${\hbox {Ge}}_2{\hbox {Sb}}$, and ${\hbox {Ge}}_2{\hbox {Bi}}$. The cohesive energies, phonon dispersions, and AIMD calculations show that, similar to ${\hbox {Sn}}_2{\hbox {Bi}}$, all of these freestanding monolayers are stable in hydrogenated form. These hydrogenated monolayers are semiconductors with wide band gaps, which are favorable for opto-electronic purposes. The ${\hbox {Si}}_2{\hbox {YH}}_2$ and ${\hbox {Ge}}_2{\hbox {YH}}_2$ structures possess indirect and direct band gaps, respectively. They represent very interesting optical characteristics, such as good absorption in the visible region and linear dichroism, which are crucial for solar cell and beam-splitting devices, respectively. Finally, the ${\hbox {Si}}_2{\hbox {SbH}}_2$ and ${\hbox {Si}}_2{\hbox {BiH}}_2$ monolayers have suitable band gaps and band edge positions for photocatalytic water splitting. Summarily, our investigations offer very interesting and promising properties for this family of binary compounds. We hope that our predictions open ways to new experimental studies and fabrication of suitable 2D materials for next generation opto-electronic and photocatalytic devices.

## Introduction

The high tower of the contemporary technology is built by blocks of silicon and germanium. Since the successful synthesize of the monolayer carbon (graphene)^1^ and discovery of its remarkable characteristics, such as high carrier mobility^2^, strong mechanical parameters^3^, and optical transparency^4^, a great inquiry for other elemental monolayers is in the agenda of many scientists around the world. The monolayers of carbon’s neighbors in group-IV, silicon and germanium (silicene and germanene) are among the most important predicted and synthesized monolayers beyond graphene^5,6^.

Unlike graphene, which is completely flat with an ${\hbox {sp}}^2$ bonding characteristics, the larger interatomic distance in silicene and germanene weakens the $\pi - \pi$ overlaps, which leads to buckled structures with ${\hbox {sp}}^2 - {\hbox {sp}}^3$ hybrid orbitals. Despite their buckled geometry, silicene and germanene share most of the important electronic features of graphene, such as Dirac cone, high Fermi velocity and carrier mobility^7,8^, with some advantages including better tunability of the band gaps^9^, stronger spin-orbit coupling^10^, and easier valley polarization^11^, which are very important for electrics, spintronics, and valleytronics.

On the other hand, monolayers of group-V elements, known as pnictogens, including phosphorene, arsenene, antimonene, and bismuthene, recently have gained much attention for their topological aspects, as well as inherent, wide, and tunable band gaps^12–15^. Generally, several allotropes are considered for these monolayers, including $\alpha$ (puckered or washboard) and $\beta$ (buckled honeycomb or graphene-like), as the most important and stable phases. For arsenene, antimonene, and bismuthene, the $\beta$-phase, and for phosphorene, the $\alpha$-phase is more stable in aspects of energetics and phonon dispersions^12,16^. The $\alpha$-phase phosphorene and arsenene possess direct band gaps, while their $\beta$ counterparts have indirect ones. On the other hand, antimonene and bismuthene respectively have indirect and direct band gaps in both phases. These band gaps are within a wide range of 0.36 (for $\alpha$-bismuthene) to 2.62 eV (for $\beta$-phosphorene)^13–15^. Moreover, phosphorene, arsenene, and bismuthene possess carrier mobilities as high as several thousand ${\text{ cm}}^2 {\text{ V}}^{-1}  {\text{s}}^{-1}$^12^. These exciting properties makes group-V monolayers very favorable candidates for optoelectronics, and photocatalytic devices.

Because of high ratio between the surface and thickness of 2D structures, effects of chemical functionalization play an important role in tuning their properties. Hence, in addition to pure elemental monolayers, 2D materials with functionalized structures gained attention for expanding the scope of realized physical aspects and enhancing potential applications. These efforts include designing and applying various types of heterostructures^17^, defections^18^, vacancies^19^, adsorptions^20^, and compounds^21,22^. Among these, binary compounds have the advantage of relatively easier fine control of the growth dynamics and more feasible fabrication. They could represent unusual atomic configuration and chemical stoichiometry^23^ which leads to extraordinary physical properties for future applications and opening ways to new researches.

As an example of group IV-V binary compound, Barreteau et al have succeed to synthesize the bulk single crystals of layered SiP, SiAs, GeP, and GeAs by melt-growth method. They showed that these layered materials all exhibit semiconducting behavior, and suggest that they can be further exfoliated into 2D structures^24^. Moreover, a number of recent theoretical works were performed on group IV-V 2D binary compounds and reported interesting results in thermoelectricity for SiX (${\hbox {X}}={\hbox {N}}$, P, As, Sb, and Bi)^25^, visible-light photohydrolytic catalysts for SiP^26^, strain-tunable electron mobility for XY (${\hbox {X}} = {\hbox {C}}$, Si, and Ge, and ${\hbox {Y}} = {\hbox {N}}$, P, and As)^27^, and ORR applications in novel fuel cells for metal (Ni, Pd, Pt, and Ru) complexes in graphene basal planes^28^.

Very recently, Gou et al. have synthesized a unique hexagonal 2D binary compound, ${\hbox {Sn}}_2{\hbox {Bi}}$, on a silicon (111) substrate which exhibits strong spin-orbit coupling and high electron-hole asymmetry^23^. In the band structure of this semiconducting monolayer, electron flat bands and free hole bands are seen which are indicatives of nearly free and strongly localized charge carriers. Moreover, this monolayer is very stable because all the Si, Bi, and Sn atoms satisfy the octet rule. These features make ${\hbox {Sn}}_2{\hbox {Bi}}$ a good candidate for nano-electronics and may result in nontrivial properties like ferromagnetism^29^ and superconductivity^30^. Furthermore, the synthesis of other group-IV-V ${\hbox {X}}_2{\hbox {Y}}$ counterparts of ${\hbox {Sn}}_2{\hbox {Bi}}$ was proposed by Gou et al^23^.

Generally, experimental synthesis of yet unknown systems can be guided by predictive theoretical first-principles calculations which distinguish stable and unstable structures correctly. In other words, theoretical predictions play an important role in progress of materials science and technology, by means of justifying the cost and effort of potential experiments. Many advances in materials science have been conducted and inspired by earlier theoretical investigations. Most of the presently well-known synthesized 2D materials, such as borophene^31^, stanene, germanene, silicene^7^, arsenene^13^, antimonene^14^, bismuthene^15^, etc. were firstly predicted by theoretical studies which brought sufficient motivations for experimental work.

Herein, inspired by the successful deposition of ${\hbox {Sn}}_2{\hbox {Bi}}$ monolayer, as well as the importance of group IV and V monolayers, we predicted a new family of binary compound monolayers with a hexagonal structure and an empirical formula of ${\hbox {X}}_2{\hbox {Y}}$, where X and Y are respectively chosen from group-IV (Si and Ge) and V (P, As, Sb, and Bi), namely ${\hbox {Si}}_2{\hbox {P}}$, ${\hbox {Si}}_2{\hbox {As}}$, ${\hbox {Si}}_2{\hbox {Sb}}$, ${\hbox {Si}}_2{\hbox {Bi}}$, ${\hbox {Ge}}_2{\hbox {P}}$, ${\hbox {Ge}}_2{\hbox {As}}$, ${\hbox {Ge}}_2{\hbox {Sb}}$, and ${\hbox {Ge}}_2{\hbox {Bi}}$. We firstly stabilize the mentioned monolayers by hydrogenation, and further check their stability by cohesive energy, molecular dynamics, and phonon dispersion analysis, and interpret their phonon modes and thermodynamical properties. Furthermore, we analyze their electronic and optical properties and discuss their potential strengths. Eventually, we consider these semiconductors for photocatalytic purposes and check their potential applications in water-splitting.

Our results suggest that these monolayers are strongly applicable in a very vast areas such as valleytronics, opto-electronics, beam-splitters, optical detectors, and water-splitters. Moreover, the structural similarity with the synthesized ${\hbox {Sn}}_2{\hbox {Bi}}$ monolayer, promises the possibility of their deposition on proper substrates and brings hopes for advances in technological devices.

## Computational details

The first-principles calculations were performed based on the density functional theory (DFT), as implemented in the Quantum Espresso package^32^. During the entire calculations, the norm-conserving (NC) pseudo-potentials with a plane wave basis set were employed to describe the electron wave functions. The generalized gradient approximation (GGA) was used with the formulation of Perdew–Burke–Ernzerhof (PBE) to describe the exchange-correlation potential^33^. Because the GGA usually underestimates the band gaps, the HSE06 hybrid functional was also used to obtain more accurate band gaps. The energy cut-off for wave function and charge density was set to 50 and 300 Ry, respectively. The Monkhorst-Pack scheme was used to sample the Brillouin zone with a $13 \times 13\times 1$ and $21 \times 21\times 1$ k-points for geometric optimization and electronic calculations, respectively. However, for the HSE calculations, the k-points was set to be $5 \times 5\times 1$. A vacuum space of 20 Å was chosen along the z-direction to prevent spurious interactions between layers in the periodic boundary condition. All the monolayers were fully relaxed with a force and stress tolerance of 10$^{-3}$ eV/Å and 10$^{-4}$ GPa, respectively. To calculate the phonon dispersion, the finite displacement method was adopted, in which a $3 \times 3\times$1 supercell with a $5 \times 5\times$1 k-point sampling was built.

To investigate the optical properties, the frequency-dependent dielectric function was calculated within the independent particle approximation (IPA) which describes single-particle excitations, as implemented in the epsilon code inbuilt in the QE package. The calculation was performed by means of self-consistent ground-state eigenvalues and eigenfunctions.

To determine the structural stability of the monolayers, their cohesive energies ($E_c$) were calculated using the equation below:

1 $$\begin{aligned} E_c=\frac{E_{sheet}-\sum _{i} n_i E_{atom-i}}{N} \end{aligned}$$

where $E_{sheet}$ and $E_{atom-i}$ stand for total energy of the sheet and the isolated atom-i with considerations of the spin polarization, respectively. *N* and $n_i$ are the numbers of total atoms and atom-i in the unit cell, respectively.

To check the thermal stability, the ab-initio molecular dynamics (AIMD) simulations were performed using NVT canonical ensemble at room temperature (300 K). The initial model was constructed by a $3 \times 3\times$1 supercell for minimizing the constraint caused by periodicity. Here, the total simulation time was set to be 4.0 ps with time steps of 2.0 fs.

## Results and discussion

### Structural stability and phonon calculations

**Table 1:** Structural parameters of the ${\hbox {X}}_2{\hbox {YH}}_2$ binary compound monolayers, including lattice constants (*a*), bond lengths ($R_{X\!X}$ and $R_{X\!Y}$), buckling heights ($\Delta$), cohesive energies ($E_c$), band gaps ($E_g$), Debye temperatures ($\theta _D$), and constant volume heat capacity in room temperature ($C_V^{300 K}$).

	*a* (Å)	$$R_{X\!X}$$ (Å)	$$R_{Y\!Y}$$ (Å)	$$\Delta $$ (Å)	$$E_c$$ (eV/atom)	$$E_g:$$ GGA, HSE (eV)	$$\theta _D$$ (K)	$$C_V^{300 K}$$ (J $$\hbox {mol}^{-1}$$$$\hbox {K}^{-1}$$)
$\hbox {Si}_2\hbox {PH}_2$	6.26	2.35	2.27	1.08	− 3.88	2.39, 3.19 (ind)	143.7	15.45
$\hbox {Si}_2\hbox {AsH}_2$	6.44	2.35	2.39	1.19	− 3.73	2.33, 3.04 (ind)	120.3	16.09
$\hbox {Si}_2\hbox {SbH}_2$	6.79	2.35	2.60	1.30	− 3.58	2.04, 2.61 (ind)	97.9	16.57
$\hbox {Si}_2\hbox {BiH}_2$	6.94	2.35	2.69	1.35	− 3.50	1.92, 2.43 (ind)	70.8	16.93
$\hbox {Ge}_2\hbox {PH}_2$	6.52	2.46	2.36	1.15	3.34	2.21, 2.88 (dir)	94.7	17.18
$\hbox {Ge}_2\hbox {AsH}_2$	6.69	2.47	2.47	1.23	− 3.24	1.80, 2.41 (dir)	83.5	17.71
$\hbox {Ge}_2\hbox {SbH}_2$	7.03	2.47	2.67	1.33	− 3.13	1.57, 2.07 (dir)	69.3	18.03
$\hbox {Ge}_2\hbox {BiH}_2$	7.18	2.48	2.75	1.38	− 3.08	1.17, 1.57 (dir)	54.9	18.22

**Figure 1:**
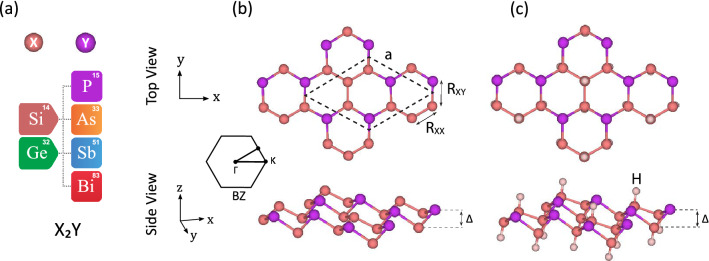
(Color online) Structural configurations of the predicted binary compound monolayers. (**a**) Table of included elements. (**b**) Top and (**c**) side view of the pure and hydrogenated monolayers. The unit cell and the corresponding Brillouin zone have also been presented.

**Figure 2:**
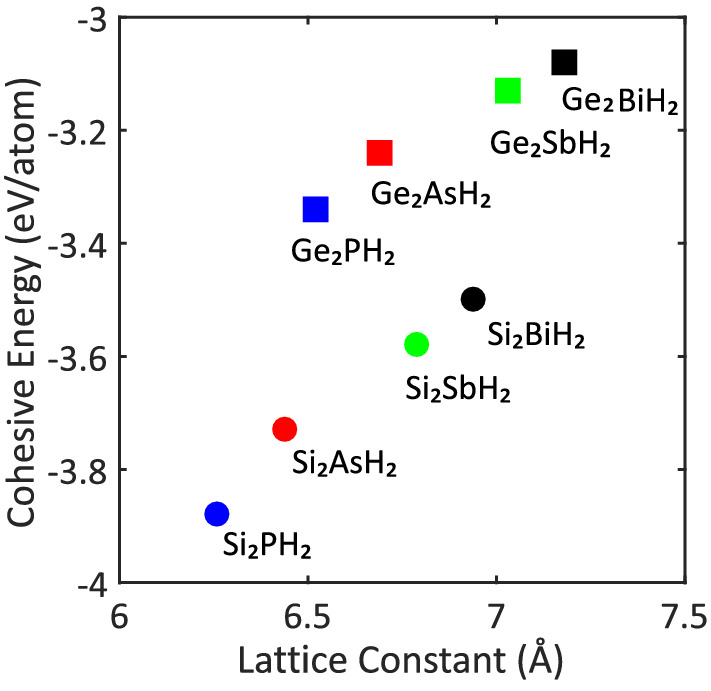
Variation of cohesive energy with lattice constant of the $\hbox {X}_2\hbox {YH}_2$ binary compound monolayers.

**Figure 3:**
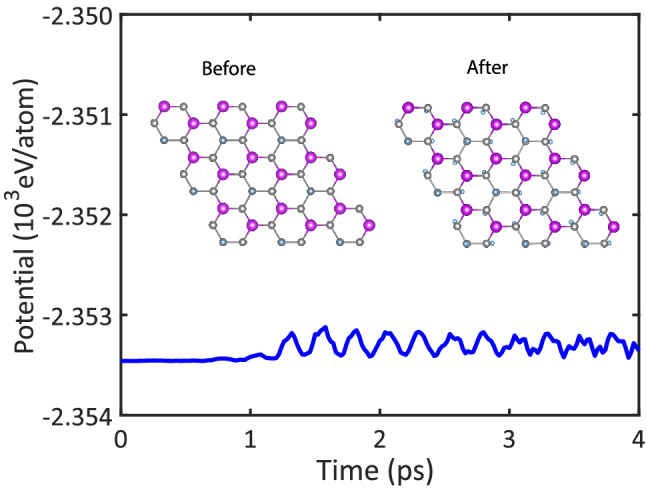
Potential energy fluctuations of the $\hbox {Ge}_2\hbox {BiH}_2$ during the AIMD simulations at 300 K. The final geometric structure at the end of 4 ps has also been shown.

**Figure 4:**
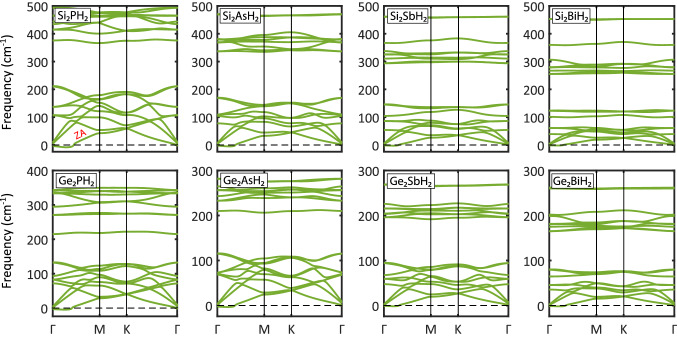
Phonon dispersion spectra of the $\hbox {X}_2\hbox {YH}_2$ binary compound monolayers. As can be seen, there are 18 phonon branches corresponding to 6 atoms in the unit cell (excluding hydrogen atoms). No considerable imaginary modes are seen, so all the structures are dynamically stable.

**Figure 5:**
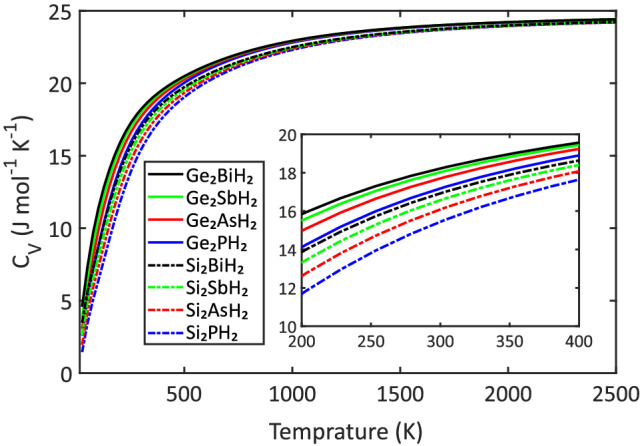
(Color online) Constant volume heat capacity ($$C_V$$) of the predicted binary compounds as a function of temperature, for one mole, and divided by the number of atoms in the unit cell (10). The $$C_V$$ is converged to $$\sim $$ 24 J $$\hbox {mol}^{-1}$$$$\hbox {K}^{-1}$$ in high-temperature limit, which is in agreement with the Debye model. Also, the heavier monolayers have greater $$C_V$$ at room temperature, which is consistent with similar studies.

The graphene-like structure was used to construct eight new binary monolayers, with a threefold-coordinated X (Si and Ge) and Y (P, As, Sb, and Bi) atoms in a hexagonal unit cell containing six atoms, as shown in Fig. 1. Through the structural optimization with the GGA-PBE exchange-correlation, the relaxed lattice constants and bond lengths were calculated in the range of 6.33 to 7.23 Å and 2.26 to 2.75 Å, respectively. All the relaxed monolayers have buckled structures with buckling heights in the range of 0.86 to 1.24 Å in which the longer atomic radius creates larger buckling heights. Moreover, all of the monolayers represent metallic electronic properties. The calculated structural parameters are available in the Supporting Information, Table S1.

The X and Y atoms have $\hbox {ns}^2$ $\hbox {np}^2$ ($\hbox {n} = 3, 4$) and $\hbox {ns}^2$ $\hbox {np}^3$ (n = 3–6) outer shell electron configurations, respectively. Therefore, when they form a threefold configuration, the octet rule only fulfills for the Y, not X atoms. Thus, these pure structures are predicted to be unstable in a freestanding configuration. Phonon dispersion analyses confirm that these monolayers are dynamically unstable (Fig. S1). The same instability has also been reported for freestanding $\hbox {Sn}_2\hbox {Bi}$ monolayer while it can be greatly stabilized by hydrogenation^34,35^.

Hydrogenation is a well-recognized technique for stabilization and tuning the physical characteristics of nano-scale systems. Many experimental studies have been done on performing different methods of hydrogenation. For example, the surface hydrogenated graphene (aka graphane) was prepared by exposure of graphene to a cold hydrogen plasma which led to the opening of a band gap and other changes in its electronic properties^36^. This success was inspired by previous theoretical predictions which conducted the experiment well into a new graphene-based structure^37,38^. In addition, several phases of borophene have been synthesized on Ag (111), Au (111), and Cu (111) substrates, which are metal and unstable in freestanding form. Eventually, a new phase of hydrogenated borophene was synthesized by thermal decomposition of sodium borohydride ($\hbox {NaBH}_4$) powders, which shows an ultra-stability and semi-conducting characteristics in the air environment^39^. This achievement was also led by theoretical predictions^40^.

In our case, the $\hbox {Sn}_2\hbox {Bi}$ monolayer was firstly synthesized on a silicon substrate^23^. Subsequently, a computational study suggested that the isolated $\hbox {Sn}_2\hbox {Bi}$ is a metal which suffers from instability due to the dangling bonds. However, it can become a stable semiconductor by use of chemical functionalization, such as surface hydrogenation^34^. By comparison, it is found that the results of hydrogenated $\hbox {Sn}_2\hbox {Bi}$ are very similar to that of $\hbox {Sn}_2\hbox {Bi}$ synthesized on the substrate. For example, in both systems, there are electron flat bands and free hole bands, which provide the possibility of having strongly localized electrons and free holes. Also, the bandgap predicted for hydrogenated $\hbox {Sn}_2\hbox {Bi}$ is 0.92 eV which agrees well with bandgap of 0.8 eV for substrate-supported monolayer revealed by angle-resolved photoemission spectroscopy measurements^23,34^. In the following, we show that the predicted $\hbox {X}_2\hbox {Y}$ monolayers, similar to $\hbox {Sn}_2\hbox {Bi}$, can be stabilized and become semiconductors by surface hydrogenation. In other words, we found an interesting analogous trend shared with $\hbox {Sn}_2\hbox {Bi}$ and its eight counterparts suggested by us. Summarily, according to similarity of $\hbox {Sn}_2\hbox {BiH}_2$ and $\hbox {Sn}_2\hbox {Bi/Si}$(111)^23,34^, we predict that the properties of the hydrogenated $\hbox {X}_2\hbox {Y}$ monolayers are also similar to possible deposited monolayers on suitable insulator substrates such as ZnS (111), SiC (111), and Si(111).

For surface hydrogenation, we investigated both single and double side hydrogenated structures, where hydrogen make bonds with X (Si and Ge) atoms, so the octet rule would be fulfilled. According to the cohesive energies, the double side hydrogenated model, having the lowest ground state energy, is predicted to be the most stable structure. Therefore, we denote the rest of the investigations to this model which is described in Fig. 1c. In the following, we further confirm their structural, thermal and dynamical stability by means of cohesive energy, molecular dynamics, and vibrational phonon analysis.

Table 1 lists the structural and electronic parameters for these monolayers. Lattice constants, bond lengths, and buckling heights are in the range of 6.26 to 7.18 Å, 2.27 to 2.75 Å, and 1.08 to 1.38 Å, respectively. As can be seen, hydrogenation causes an increase in buckling heights and a decrease in lattice constants for all the monolayers, which is due to the strong bonds between H and X atoms. Similar behaviors have also been reported for hydrogenation and fluorination of penta-graphene^41^, silicene^42^, germanene^43^, and stanene^44^.

We have also performed the ab-initio molecular dynamics (AIMD) simulations to verify the thermal stability of the $\hbox {X}_2\hbox {YH}_2$ binary compounds. Figure 3 exhibits the fluctuations of potential energy and evolutions of geometric structure of the $\hbox {Ge}_2\hbox {BiH}_2$ monolayer during the simulations at 300 K. As can be seen, the potential energy oscillates with an extent of less than 0.4 eV/atom, and no obvious structural distortions are found, indicating that the $\hbox {Ge}_2\hbox {BiH}_2$ is thermally stable at 300 K. The thermal stability of the $\hbox {Ge}_2\hbox {BiH}_2$ guarantees stability of all the predicted structures because it has the highest cohesive energy among them (see Fig. 2). Indeed, this suggests that the $\hbox {X}_2\hbox {YH}_2$ binary compounds can be realized experimentally at room temperature (Fig. 3).

To further confirm the stability of the hydrogenated monolayers, the phonon dispersion spectra were calculated and displayed in Fig. 4. It is clear that there is no imaginary frequency in the whole Brillouin zone, which confirms that these freestanding monolayers are dynamically stable. The spoon-shaped curves near the $\Gamma$ point do not mean instability, but they are signatures of the flexural acoustic modes, which are usually hard to converge in 2D sheets. These soft modes are also found in other analogous systems^45,46^.

All the phonon spectra have rather similar trends, which mean similar bonding. Also, it is clear that the maxima of acoustic modes decline with going down in group IV and V where $\hbox {Si}_2\hbox {PH}_2$ and $\hbox {Ge}_2\hbox {BiH}_2$ display the highest (100 $\hbox {cm}^{-1}$) and lowest (38 $\hbox {cm}^{-1}$) peaks. Based on these maxima, the Debye temperatures are obtained by $\theta _D=h\nu _m/K\!_B$^47^, where *h* and $K\!_B$ are the Planck and Boltzmann constants, respectively. The calculated temperatures are in the range of 143 to 54 K (listed in Table 1) which are lower than graphene (2266 K), silicene (798 K), phosphorene (206 K), arsenene (170 K), and comparable to antimonene (101 K), bismuthene (50 K), and stanene (72 K)^47–50^. Such low Debye temperatures and large buckling heights, which are indicatives of low lattice thermal conductivity, may bring hope for these monolayers to be suitable candidates for thermoelectric applications.

Interestingly, the slope of the parabolic out-of-plane acoustic mode (ZA) near the $\Gamma$ point (specified in Fig. 4) decreases with increasing of the average atomic mass of the monolayers. This will bring a slower phonon group velocity, subsequently lower lattice thermal conductivity, and stronger anharmonicity, especially for $\hbox {Si}_2\hbox {BiH}_2$, $\hbox {Ge}_2\hbox {AsH}_2$, $\hbox {Ge}_2\hbox {SbH}_2$, and $\hbox {Ge}_2\hbox {BiH}_2$. It is worth noting that the ZA mode has a high contribution to the phonon transport^51^. On the other hand, the hybridization of the optical and acoustic phonon branches increases the phonon scattering which reveals low phonon transport. These behaviors represent the possible potential of $\hbox {X}_2\hbox {YH}_2$ monolayers in thermoelectricity.

According to Eq. (1), the more negative values for cohesive energies suggest more structural stability for the monolayers. As shown in Table 1, the cohesive energies vary from − 3.88 eV/atom for $\hbox {Si}_2\hbox {PH}_2$ to − 3.08 eV/atom for $\hbox {Ge}_2\hbox {BiH}_2$ which indicates that all of the monolayers are stable. In fact, the structures represent more stability when the atoms are lighter. By comparison, one can easily realize that all the predicted monolayers are more stable than the hydrogenated $\hbox {Sn}_2\hbox {Bi}$ ($\hbox {Sn}_2\hbox {BiH}_2$), which was discussed in our previous study to have a cohesive energy of − 2.95 eV/atom^35^. Also, the $\hbox {Si}_2\hbox {SbH}_2$, $\hbox {Si}_2\hbox {BiH}_2$, $\hbox {Ge}_2\hbox {SbH}_2$, and $\hbox {Ge}_2\hbox {BiH}_2$ monolayers are more stable than SiSb (− 3.50 eV/atom), SiBi (− 3.31 eV/atom), GeSb (− 3.12 eV/atom), and GeBi (− 2.98 eV/atom) binary compounds, respectively. The rest have appreciable cohesive energies comparable to SiP (− 4.19 eV/atom), SiAs (− 3.85 eV/atom), GeP (− 3.60 eV/atom), and GeAs (− 3.36 eV/atom)^52^. All the mentioned cohesive energies above were calculated through GGA-PBE functional. For a better comparison between cohesive energies of the predicted binary compounds, please pay attention to Fig. 2.

Phonon dispersion is also a key to calculate thermodynamic properties of a system. For example, the constant volume heat capacity, $C_V$ is defined as^53^:

2 $$\begin{aligned} C_V=\sum _{s,q}K\!_B\left( \frac{\hslash \omega _s(q)}{K\!_BT}\right) ^2 \frac{exp(\hslash \omega _s(q)/K\!_BT)}{\left( exp(\hslash \omega _s(q)/K\!_BT)-1\right) ^2} \end{aligned}$$

where $\hslash$ is the reduced Planck’s constant, and $\omega _s(q)$ is the frequency of the *s* phonon branch at the *q* point. According to the Debye model, in the high-temperature limit, i.e. $K\!_BT \gg \hslash \omega$, the heat capacity simply approaches to the classical Dulong-Petit results, which is $3N\!M\!K\!_B$, where *N* is the number of atoms in the unit cell and *M* is the number of unit cells in a crystal ($\approx$ 24.94 J $\hbox {mol}^{-1}$ $\hbox {K}^{-1}$ for one mole of a mono-atomic solid)^54^. Figure 5 exhibits the $C_V$ calculated for the hydrogenated binary compounds as a function of temperature (one mole, divided by the number of atoms in the unit cell) which was calculated by use of the phonon dispersion spectra. As it is clear, the $C_V$ is converged to $\sim$ 24 J $\hbox {mol}^{-1}$ $\hbox {K}^{-1}$ in high-temperature limit, which is in good agreement with the Debye model.

Moreover, the $C_V$ for $\hbox {Si}_2\hbox {PH}_2$, $\hbox {Si}_2\hbox {AsH}_2$, $\hbox {Si}_2\hbox {SbH}_2$, $\hbox {Si}_2\hbox {BiH}_2$, $\hbox {Ge}_2\hbox {PH}_2$, $\hbox {Ge}_2\hbox {AsH}_2$, $\hbox {Ge}_2\hbox {SbH}_2$, and $\hbox {Ge}_2\hbox {BiH}_2$, at room temperature (300 K) are 15.45, 16.09, 16.57, 16.93, 17.18, 17.71, 18.03, and 18.22 J $\hbox {mol}^{-1}$ $\hbox {K}^{-1}$, respectively (see Table 1). Despite the importance of the $C_V$ in the understanding of thermal properties, it has not gained sufficient attention in 2D materials so far. To the best of our probe, some examples of similar calculations are: 23.1 (TiSeS), 22.7 (TiTeS), 22.5 (TiSeTe), 17.5 ($\hbox {CuTe}_2\hbox {O}_5$), 11.5 (borophene) J $\hbox {mol}^{-1}$ $\hbox {K}^{-1}$^53,55,56^, which are comparable with our results. It is provable that heavier materials have a greater $C_V$ at room temperature, i.e. they are more resistant to temperature increase. Therefore, one may conclude that compared with borophene, all of the predicted binary compounds, and compared with $\hbox {CuTe}_2\hbox {O}_5$, the $\hbox {Ge}_2\hbox {AsH}_2$, $\hbox {Ge}_2\hbox {SbH}_2$, and $\hbox {Ge}_2\hbox {BiH}_2$ monolayers are better electronic devices in the aspects of not overheating. With confirming the structural stability and discussing the thermodynamical characteristics, now we turn our attention into the electronic properties of the predicted binary compounds.

### Electronic properties

**Figure 6:**
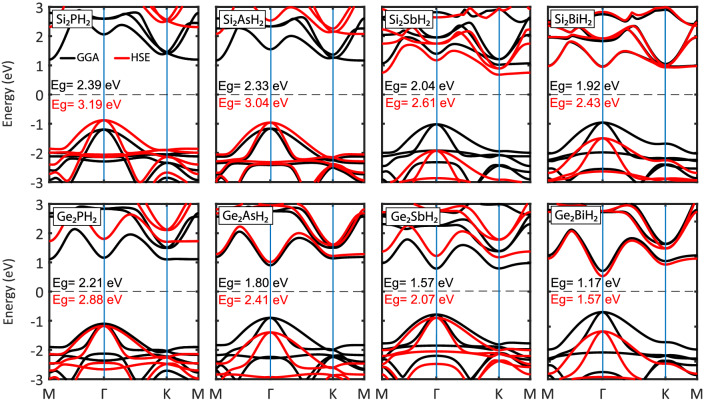
(Color online) Energy band structures of the $\hbox {X}_2\hbox {YH}_2$ binary compound monolayers along the main high symmetry k-points at the GGA (black lines) and HSE06 (red lines) levels together with the band gap values. The Fermi levels were shifted to zero.

The electronic band structures of $\hbox {X}_2\hbox {YH}_2$ binary compound monolayers have been presented at the GGA and HSE06 levels in Fig. 6. As can be seen, all the monolayers are semiconductors. The $\hbox {Ge}_2\hbox {YH}_2$ monolayers have direct band gaps at the $\Gamma$ point. In contrast, the $\hbox {Si}_2\hbox {YH}_2$ monolayers have indirect band gaps where their valence band maxima (VBM) are located at the $\Gamma$ point and their conduction band minima (CBM) are located at the M (for $\hbox {Si}_2\hbox {PH}_2$ and $\hbox {Si}_2\hbox {AsH}_2$) and K (for $\hbox {Si}_2\hbox {SbH}_2$ and $\hbox {Si}_2\hbox {BiH}_2$) points, which are identical at both GGA and HSE levels. The band gaps predicted at the HSE level are in the range of 1.57 to 3.19 eV, where $\hbox {Si}_2\hbox {PH}_2$ and $\hbox {Ge}_2\hbox {BiH}_2$ exhibit the largest and smallest values, respectively (see Table 1). It is obvious that the band gaps decrease regularly with increasing the average atomic mass, which is rather common in 2D semiconductors^45,52^. For example, in group V binary compound monolayers, studied by Zhang et al, the PAs and SbBi monolayers indicate the largest (2.55 eV) and smallest (1.41 eV) band gaps, respectively. In more details, the reported band gaps are in the order of PAs > PSb > PBi > AsBi > SbBi^57^.

All the calculated band structures demonstrate parabolic valence bands centered at the $\Gamma$ point which provides high hole conductivity. Among these, the $\hbox {Ge}_2\hbox {YH}_2$ structures have parabolic conduction bands centered at $\Gamma$ point, which indicate free electrons, while the $\hbox {Si}_2\hbox {YH}_2$ structures have nearly flat conduction bands along the K − M direction, which are signatures of localized electrons. In other words, the $\hbox {Si}_2\hbox {YH}_2$ structures have both free and strongly localized charge carriers like the $\hbox {Sn}_2\hbox {Bi}$ monolayer deposited on the silicon substrate^23^.

This high electron-hole asymmetry enforces the materials to exhibit completely different optical and thermoelectric behavior in the n-type and p-type doping. In addition, all the monolayers have some conduction band extrema (CBE) near the CBM at high symmetry points M, $\Gamma$, and K which may be favorable for an n-type Seebeck coefficient^58^. These CBEs may approach each other by mechanical strain to achieve band convergence^59^. The band convergence improves electrical conductivity without affecting other transport coefficients. These features would make the $\hbox {X}_2\hbox {YH}_2$ monolayers possible candidates for thermoelectric applications.

**Figure 7:**
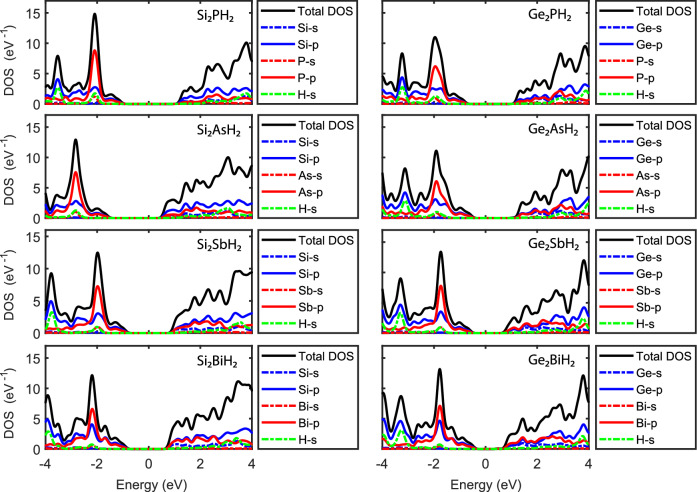
(Color online) Total and partial density of states of the $\hbox {X}_2\hbox {YH}_2$ binary compound monolayers at the GGA level. The Fermi levels were shifted to zero.

**Figure 8:**
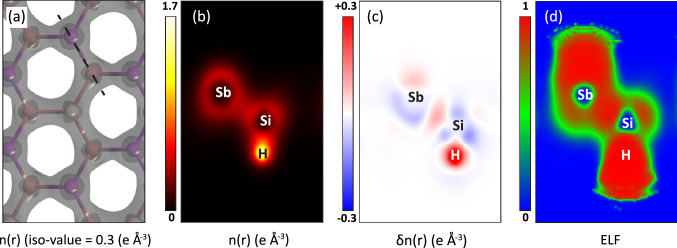
(Color online) Iso-surface and cut plane of the $\hbox {Si}_2\hbox {SbH}_2$ monolayer: (**a**) Iso-surface of electron density with an iso-value of 0.3 *e*Å$^{-3}$. (**b**) Cross-section cut plane of electron density, (**c**) electron difference density, and (**d**) electron localization function (ELF) along the black dashed line in (**a**). The color bar next to the sub-figures denotes the scope of each quantity. In (**c**), the red and blue colors show electron accumulation and depletion, respectively.

We also took into account the spin-orbit coupling (SOC) interaction in the calculation of the GGA band structures (SOGGA) as presented in Fig. S2. It can be seen that consideration of the SOC, more or less, terminates the degeneracy between energy bands and narrows the band gaps. Due to the stronger spin-orbit interactions for heavier atoms, the band splitting increases as the compounds are heavier.

Summarily, the effect of SOC on the band gaps is smaller than 0.3 eV for most of the monolayers, except for relatively heavy $\hbox {X}_2\hbox {BiH}_2$ ($\hbox {Si}_2\hbox {BiH}_2$ & $\hbox {Ge}_2\hbox {BiH}_2$) which have SOGGA band gaps approximately 0.5 eV smaller than that of GGA. Overall, for its small influence on most of the monolayers, the SOC was not considered for the rest of our calculations. Figure 7 shows the total and orbital projected density of states of the hydrogenated binary monolayers. It can be seen that in the whole energy range, the p orbitals are dominant and the s orbitals have negligible proportions in the electronic characteristics, which was predictable according to the electronic arrangement of the contained atoms. This domination have been reported for other group IV and V 2D structures^13,14,60,61^. As it is clear, for all the monolayers, the Y-p orbitals are dominant in the valance bands, and major peaks around − 2 eV are raised by them. These are attributed to the rather flat bands around − 2 eV in the band structures (see Fig. 6). On the other hand, the conduction bands are slightly dominated by Si atom for the Si contained structures, while for the Ge contained ones, Ge-p and Y-p orbitals share rather equal proportions of the conduction band states.

Also, more or less, we see an overlapping of DOS of X-p and Y-p orbitals near the Fermi energy for all the monolayers, which are signatures of strong covalent bonds between the atoms, due to the orbitals hybridization. As can be seen, orbitals hybridization is rather similar for all the compounds in the valance bands, but in the conduction bands, it is not significant for $\hbox {Si}_2\hbox {PH}_2$ and $\hbox {Si}_2\hbox {AsH}_2$. More interestingly, in the Ge contained compounds, the Y-s orbitals also participate in the hybridization. Orbitals hybridization between different atoms was also reported for other structures such as $\hbox {Sn}_2\hbox {Bi}$, $\hbox {C}_3\hbox {N}$, $\hbox {C}_3\hbox {P}$, and $\hbox {C}_3\hbox {As}$ compounds^34,62^.

Moreover, the H atoms have a very limited contribution in the DOS, which means that electrons are strongly bound to them and do not construct many states in the valance and conduction bands. Namely, a very small hybridization with Y-s orbitals, and no interfere with X orbitals is seen, which suggests ionic bonds between the H and X atoms.

To shed more light on the electronic properties and bonding mechanism of the compounds, electron density (*n*(*r*)), electron difference density ($\delta n(r)$), and electron localization function (ELF) were calculated at the GGA level. Our calculations display that all the monolayers have similar characteristics, therefore, we only present the analyses for $\hbox {Si}_2\hbox {SbH}_2$ monolayer, as a representative, in Fig. 8. Analyses for the rest of the monolayers are available in Fig. S3–S5. It is clear from Fig. 8a, that the lattice has a minimum uniform electron density of about 0.3 e Å$^{-3}$ which exhibits an in-plane isotropic lattice in aspects of electronic characteristics. It is obvious from *n*(*r*) and $\delta n(r)$ (Fig. 8b,c) that there is a gentle electron accumulation between Sb and Si atoms. Moreover, the ELF (Fig. 8d) indicates a high localization between these atoms. Therefore, one could conclude that the Sb and Si atoms share electrons mutually and make covalent bonds.

Meanwhile, there is a high electron density and accumulation on the H, with significant electron depletion around Si atoms. Besides, the ELF displays the highest localization on the H and a low localization around the Si atoms. Therefore, it is deducible that the H atoms make ionic bonds with Si atoms. This approves our discussion about the low contribution of H related electrons in the density of states. Also, the strong ionic bonds make sense about the stability of the monolayers after hydrogenation. In other words, the hydrogenation somehow plays the role of a substrate for the originally unstable pristine monolayers and stabilizes them.

### Optical properties

**Figure 9:**
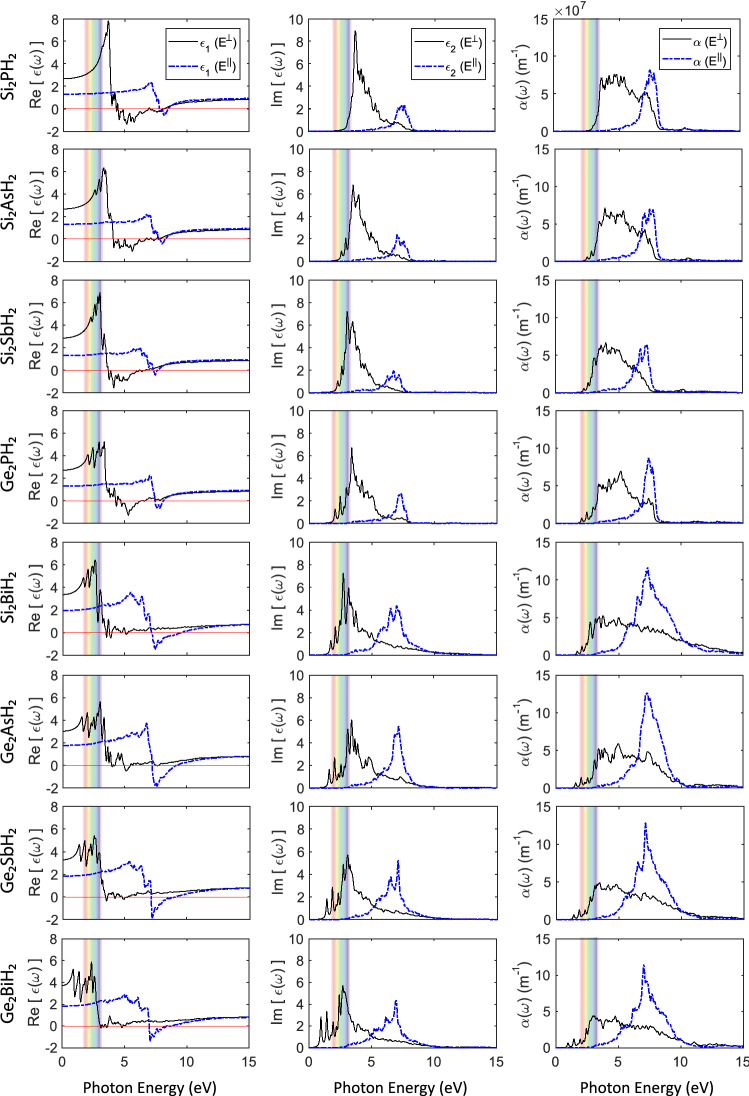
Optical properties of the $\hbox {X}_2\hbox {YH}_2$ binary compound monolayers, including the real and imaginary parts of the dielectric function ($\epsilon _1$ and $\epsilon _2$), and absorption coefficient ($\alpha$), for the perpendicular ($\hbox {E}^\perp$) and parallel ($\hbox {E}^\parallel$) polarizations.

**Table 2:** Optical parameters of the $\hbox {X}_2\hbox {YH}_2$ binary compounds, for perpendicular and parallel polarizations.

		$\hbox {E}^\perp$			$\hbox {E}^\parallel$		
		Metallic Range (nm)	$E (\epsilon _2^{peak})$ (eV)	High Abs. Range (nm)	Metallic Range (nm)	$E (\epsilon _2^{peak})$ (eV)	Sig. $\alpha$ Rng. (nm)
**Group-A**							
$\hbox {Si}_2\hbox {PH}_2$		280–155	3.74	375–150	–	7.69	195–145
$\hbox {Si}_2\hbox {AsH}_2$		310–165	3.53	395–155	–	7.06	200–150
$\hbox {Si}_2\hbox {SbH}_2$		340–200	3.05	465–170	–	6.77	220–160
$\hbox {Ge}_2\hbox {PH}_2$		290–195	3.4	430–155	–	7.37	210–155
**Group-B**							
$\hbox {Si}_2\hbox {BiH}_2$		–	2.72	525–100	175–130	7	250–125
$\hbox {Ge}_2\hbox {AsH}_2$		–	3.38	450–135	170–140	7.15	250–125
$\hbox {Ge}_2\hbox {SbH}_2$		–	3.05	495–120	175–135	7.07	255–120
$\hbox {Ge}_2\hbox {BiH}_2$		–	2.72	530–120	180–140	6.98	270–125

Metallic Range Stands for the range in which the real part of the dielectric function has negative values, and the monolayer is metallic. E ($\epsilon _2^{peak}$) represents the energy in which the imaginary part of the dielectric function has the major peak. Sig. $\alpha$ Rng. shows the range in which the optical absorption coefficient has significant values (e.g. $\alpha \ge 10^{7} m^{-1}$). The mentioned ranges are rounded to the nearest 5 nm for more clarity.

High optical absorption in 2D materials brings hopes for energy harvesting purposes such as solar cells. Moreover, linear dichroism is a phenomenon widely reported for 2D materials, which is the difference between optical absorption for light beams polarized parallel and perpendicular to an orientation axis, and is a key element for interesting optical applications such as beam splitters, LCDs, half-mirrors, etc.^63,64^. For instance, it is reported that Sb and As monolayers have optical absorption edges near $\sim$ 2 and $\sim$ 3 eV , for perpendicular and parallel polarizations, respectively^64^.

As mentioned in the previous section, the $\hbox {X}_2\hbox {YH}_2$ binary compounds were predicted to have hopeful signs of optical potentials, such as wide band gaps in the range of visible light. In this section, we calculate and discuss the optical properties of these monolayers to extract more physical insights and possible applications.

The optical properties are associated with the interactions between light, electrons, and ions in the materials, which should be explained through the complex dielectric function, $\epsilon (\omega )=\epsilon _1(\omega )+i\epsilon _2(\omega )$. Based on Fermi’s golden rule, one can derive the imaginary part of the dielectric function as below^65^:

3 $$\begin{aligned} \epsilon _2(\omega )=\frac{4\pi ^2e^2}{m^2\omega ^2}\sum _{C,V}\left| P_{C,V} \right| ^2\delta \left( E_C - E_V - \hslash \omega \right) \end{aligned}$$

where *e* is the electron charge, *m* is the electron effective mass, *P* is the momentum transition matrix, and *E* is the electron energy level. Moreover, *C* and *V* indices stand for conduction and valance bands, respectively. No need to explain, $\delta (x-x_0)$ is the Dirac delta function, which ensures conversion of energy during electron transitions from band to band. This means that every excited state has an infinite lifetime, i.e. is stationary^66^. Subsequently, the real part can be calculated through Kramer-Kronig relation:^67^

4 $$\begin{aligned} \epsilon _1(\omega )=1+\frac{2}{\pi }\int _{0}^{\infty }\frac{\omega '\epsilon _2(\omega ')}{\omega '^2-\omega ^2}d\omega \end{aligned}$$

Moreover, based on the real and imaginary parts, the optical absorption coefficient, $\alpha (\omega )$, is calculated through:

5 $$\begin{aligned} \alpha (\omega )=2\frac{\omega }{c}\sqrt{\frac{\sqrt{\epsilon _1^2+\epsilon _1^2}-\epsilon _1}{2}} \end{aligned}$$

where *c* is the speed of light.

For the isotropy of the monolayers in the xy plane, there is no significant difference between xx and yy polarizations, therefore the calculations were performed for polarized radiations, parallel ($\hbox {E}^\parallel$) and perpendicular ($\hbox {E}^\perp$) to the incidence direction (z-direction). Figure 9 shows the calculated optical properties of the $\hbox {X}_2\hbox {YH}_2$ binary compounds, including real and imaginary parts of the dielectric function ($\epsilon _1$ and $\epsilon _2$), and the absorption coefficient ($\alpha$), for both polarizations. Interestingly, the predicted monolayers can be separated into two groups, group-A, including $\hbox {Si}_2\hbox {PH}_2$, $\hbox {Si}_2\hbox {AsH}_2$, $\hbox {Si}_2\hbox {SbH}_2$, and $\hbox {Ge}_2\hbox {PH}_2$, and group-B including $\hbox {Si}_2\hbox {BiH}_2$, $\hbox {Ge}_2\hbox {AsH}_2$, $\hbox {Ge}_2\hbox {SbH}_2$, and $\hbox {Ge}_2\hbox {BiH}_2$. The materials in each group exhibit similar properties, which will be discussed in detail.

As we know, negative values in the real part of the dielectric function stand for metallic reflectivity^68^. As it is clear in Fig. 9 (left panel), group-A monolayers have significant negative values in the real part of the dielectric function within $\sim$ 3.6 to 8 eV ($\sim$ 345 to 155 nm) in the UV region, for perpendicular polarized radiation ($\hbox {E}^\perp$). On the contrary, group-B monolayers have significant negative values within $\sim$ 6.8 to 9.5 eV ($\sim$ 180 to 130 nm), for parallel polarized radiation ($\hbox {E}^\parallel$). In other words, group-A and group-B materials are metallic for $\hbox {E}^\perp$ and $\hbox {E}^\parallel$ UV radiation, within the mentioned ranges, respectively. This means that group-A and group-B monolayers have a good complement in blocking the UV radiation and may be used together as a heterostructure for more efficient beam splitting, and UV protection purposes. Compared with the Si and Ge monolayers, which have been reported to have a metallic characteristics in the range of $\sim$ 4 to 7 eV (310 to 177 nm) and $\sim$ 0 to 4 eV ($\infty$ to 310 nm), respectively^44,69^, most of the predicted $\hbox {X}_2\hbox {YH}_2$ binary compounds have better UV blocking. For more details, please see Table 2.

The imaginary part of the dielectric function and the absorption coefficient are bound to each other and should be analyzed together. Based on the band to band transition theory, the peaks in the imaginary part of the dielectric function are concerned with energy absorption and direct transitions of electrons between bands below and above the Fermi level. As can be seen in Fig. 9 (middle panel), all the monolayers have major peaks around $\sim$ 3.5 and $\sim$ 7 eV for $\hbox {E}^\perp$ and $\hbox {E}^\parallel$ polarizations, respectively.

Moreover, in group-A monolayers, the $\hbox {E}^\perp$ peaks are much stronger than the $\hbox {E}^\parallel$ peaks, whereas, in group-B monolayers, they are relatively equal. This would be representative of the difference, and equality of significant absorption ranges ($\alpha \ge 10^{7} m^{-1}$) between $\hbox {E}^\perp$ and $\hbox {E}^\parallel$ polarizations, for group-A and group-B monolayers, respectively. In other words, as it is shown in Fig. 9 (right panel), group-B monolayers have relatively wider significant absorption ranges for $\hbox {E}^\parallel$ polarizations, which is due to the stronger $\hbox {E}^\parallel$ peaks in the imaginary part of the dielectric function.

The widest significant absorption range belongs to $\hbox {Si}_2\hbox {BiH}_2$, which is in the range of 2.36 to 12.4 eV (525 to 100 nm). For comparison, it should be noted that the Si and Ge monolayers have significant optical absorption in the range of $\sim$ 3.5 to 5 eV (354 to 248 nm) and $\sim$ 3 to 6 eV (413 to 206.6 nm), respectively^44,69^. Our calculations show that most of the predicted compounds have greatly wider significant absorption ranges. For more details about the optical properties, please note to Table 2.

Summarily, one can conclude that group-A monolayers, having stronger linear dichroism, have more potential applications in beam splitting, and group-B monolayers, having a wider absorption range for both polarizations, are more favorable for energy harvesting systems and solar cells. It should be added that three of group-B monolayers, namely $\hbox {Ge}_2\hbox {AsH}_2$, $\hbox {Ge}_2\hbox {SbH}_2$, and $\hbox {Ge}_2\hbox {BiH}_2$ have direct and wide band gaps, which makes them even more ideal for this purpose.

### Photocatalytic properties

**Figure 10:**
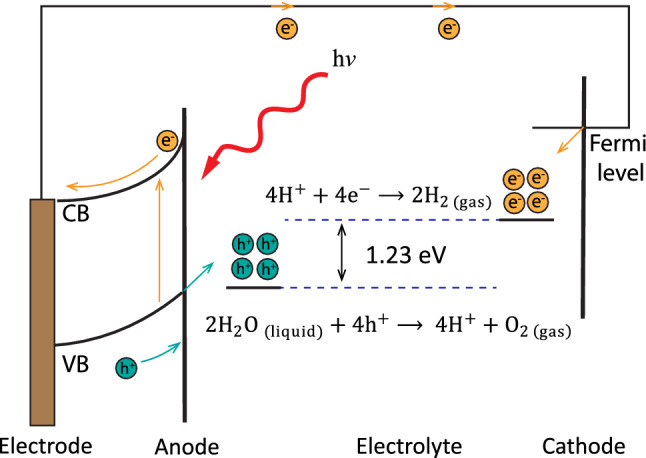
A schematic of photocatalytic water splitting process.

Water splitting is a chemical reaction in which the water molecule is broken down into oxygen and hydrogen. This process has attracted much attention because of clean, inexpensive, and environment friendly production of hydrogen. One of the well-known methods for water splitting is photocatalysis by use of a semiconductor sheet and solar energy^70^. The general chemical formula for this reaction is presented as^71^:

The first half reaction shows the water oxidation at the anode and the second one indicates the water reduction at the cathode. The overall process results in production of hydrogen and oxygen gases as illustrated in Fig. 10. A semiconductor could be a potential photocatalyst for water splitting if the CBM energy is higher than the reduction potential of $\hbox {H}^+/\hbox {H}_2$, and the VBM energy is lower than the oxidation potential of $\hbox {O}_2/\hbox {H}_2\hbox {O}$^72^. It should be noted that there are not many photocatalysts that meet all of the requirements, so far. Therefore, finding a suitable candidate semiconductor for this purpose is a crucial challenge, that we are going to face in this section.

6 $$ \begin{aligned}    & 2{\text{H}}_{2} {\text{O}}_{{({\text{Liquid}})}}  + 4{\text{h}}^{ + }  \to 4{\text{H}}^{ + }  + {\text{O}}_{{2({\rm {gas}})}}  \\     & 4{\text{H}}^{ + }  + {\text{e}}^{ - }  \to 2{\text{H}}_{{2({\rm {gas}})}}  \\  \end{aligned}  $$

**Figure 11:**
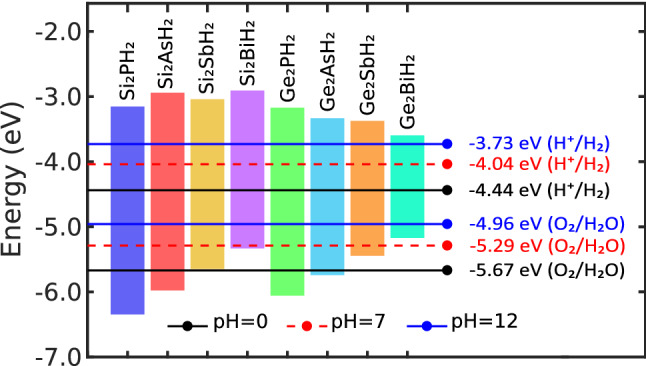
(Color online) Band edge positions of $\hbox {X}_2\hbox {YH}_2$ binary compound monolayers for photocatalytic water splitting, calculated at the HSE06 level. The redox potentials of water splitting reaction have been specified at the $\hbox {pH}=0$ (black solid lines), $\hbox {pH}=7$ (red dashed lines), and $\hbox {pH}=12$ (blue solid lines).

Due to dependency of the reduction/oxidation (known as redox) potentials to the pH, these potentials were adopted at $\hbox {pH}=0$, 7, and 12, similar to the previous studies^73,74^. In Fig. 11, the HSE06 band edges of the $\hbox {X}_2\hbox {YH}_2$ monolayers have been presented with respect to the vacuum level. As can be seen, at $\hbox {pH}=0$, the $\hbox {X}_2\hbox {PH}_2$ and $\hbox {X}_2\hbox {AsH}_2$ monolayers have suitable band edge for water splitting reaction while at $\hbox {pH}=12$, all the monolayers are eligible. However, this reaction usually occurs in a neutral environment ($\hbox {pH}=7$). At this pH, all the monolayers except $\hbox {Ge}_2\hbox {BiH}_2$ satisfy the condition of the band edge position.

As suggested by Zhang et al, materials with indirect band gaps are more desirable for photocatalytic activity^75^, therefore $\hbox {Si}_2\hbox {YH}_2$ monolayers will react better than $\hbox {Ge}_2\hbox {YH}_2$ ones. On the other hand, the band gap value should be smaller than 3 eV for enhancing the visible light absorption^76,77^, therefore the $\hbox {Si}_2\hbox {PH}_2$ and $\hbox {Si}_2\hbox {AsH}_2$ monolayers, having large band gaps for visible light, cannot produce high efficiency for electron-hole generation and accordingly for water splitting. Summarily, the $\hbox {Si}_2\hbox {SbH}_2$ and $\hbox {Si}_2\hbox {BiH}_2$ monolayers are very promising candidates for water splitting.

## Conclusion

In summary, using first-principles calculations, for the first time, we have proposed a new family of two-dimensional binary compounds with an empirical formula of $\hbox {X}_2\hbox {Y}$, where X and Y belong to groups IV (Si and Ge) and V (P, As, Sb, and Bi), respectively. Different from their pure structures, the hydrogenated ($\hbox {X}_2\hbox {YH}_2$) monolayers exhibit a very high stability according to cohesive energy, phonon dispersion analysis, and AIMD simulations. We have obtained many interesting physical properties by computing the electrical, optical, and photocatalytic behavior of these monolayers. Our calculations disclose that all of the monolayers are semiconductors with band gaps in the range of 1.57 to 3.19 eV. The optical results reveal that $\hbox {Si}_2\hbox {PH}_2$, $\hbox {Si}_2\hbox {AsH}_2$, $\hbox {Si}_2\hbox {SbH}_2$, and $\hbox {Ge}_2\hbox {PH}_2$ monolayers have potential applications in beam splitting, and $\hbox {Si}_2\hbox {BiH}_2$, $\hbox {Ge}_2\hbox {AsH}_2$, $\hbox {Ge}_2\hbox {SbH}_2$, and $\hbox {Ge}_2\hbox {BiH}_2$ monolayers are more favorable for energy harvesting systems and solar cells. Besides, the $\hbox {Si}_2\hbox {SbH}_2$ and $\hbox {Si}_2\hbox {BiH}_2$ monolayers were found to have suitable band gaps and band edge positions for photocatalytic water splitting. Our results suggest the binary monolayers of group IV-V for uses in nano-electronic and optoelectronic applications, and propose them for further experimental works. Finally, we predict that the reported properties for $\hbox {X}_2\hbox {YH}_2$ monolayers would be also similar to possible deposited $\hbox {X}_2\hbox {Y}$ monolayers on a proper substrate such as Si (111), ZnS (111), and SiC (111).

## Supplementary information

Supplementary material 1
