# Supplementary material for: Prediction of hydrogenated group IV–V hexagonal binary monolayers
Source: Sci Rep. 2020 Sep 11;10:14963. doi: 10.1038/s41598-020-71766-5 (PMC7486411; doi:10.1038/s41598-020-71766-5)
Supplement: Supplementary file 1 — Supplementary material 1 [file 41598_2020_71766_MOESM1_ESM.pdf]

Supporting Information for:

Prediction of hydrogenated group IV-V hexagonal binary monolayers

Mohammad Ali Mohebpour,[1] Shobair Mohammadi Mozvashi,[1] Sahar Izadi Vishkayi,[2] and Meysam Bagheri Tagani[1,*]

[1] *Department of physics, Computational Nanophysics Laboratory (CNL), University of Guilan, P.O.Box 41335-1914, Rasht, Iran.*

[2] *School of Physics, Institute for Research in Fundamental Sciences (IPM), P. O. Box 19395-5531, Tehran, Iran.*

TABLE S1. Structural parameters for the pure X_2Y binary compound monolayers, including lattice constants (a), bond lengths (R_{XX} & R_{XY}), and buckling heights ($\varDelta$).

	a (Å)	R_{XX} (Å)	R_{YY} (Å)	$\varDelta$ (Å)
Si_2P	6.33	2.31	2.26	0.86
Si_2As	6.51	2.31	2.38	1
Si_2Sb	6.85	2.32	2.59	1.13
Si_2Bi	6.99	2.32	2.68	1.21
Ge_2P	6.59	2.48	2.36	0.89
Ge_2As	6.77	2.48	2.47	1.02
Ge_2Sb	7.09	2.48	2.67	1.17
Ge_2Bi	7.23	2.47	2.75	1.24

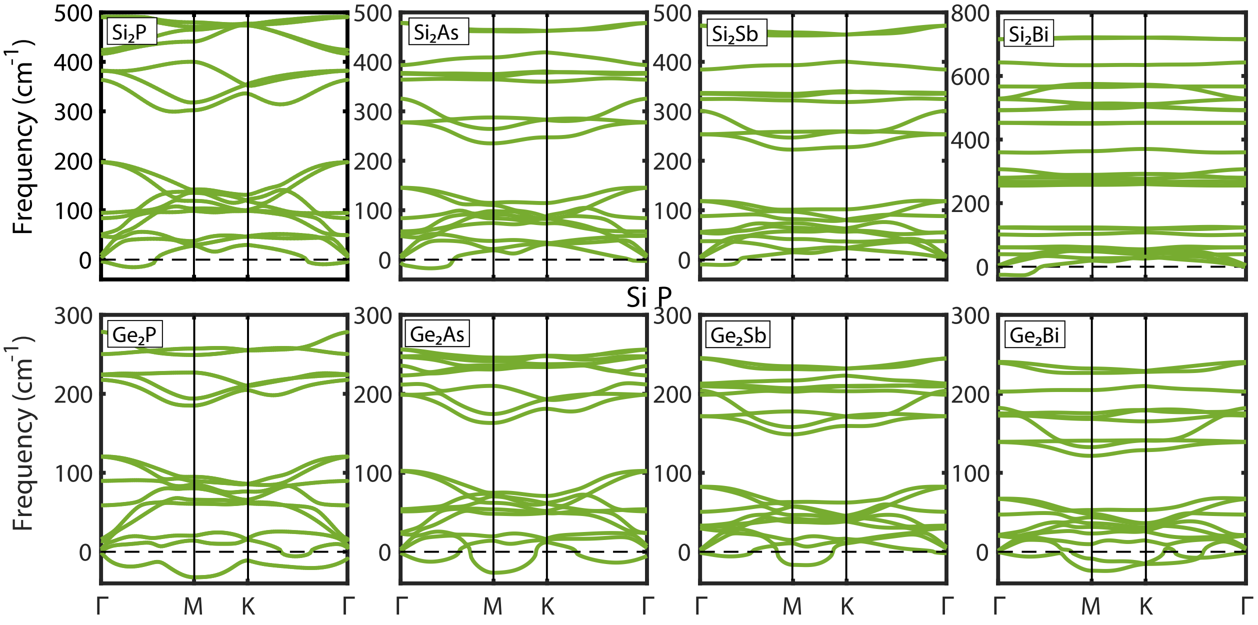

FIG. S1. Phonon dispersion calculations of the pure X_2Y binary compound monolayers. All the structures are dynamically unstable.

* m_bagheri@guilan.ac.ir

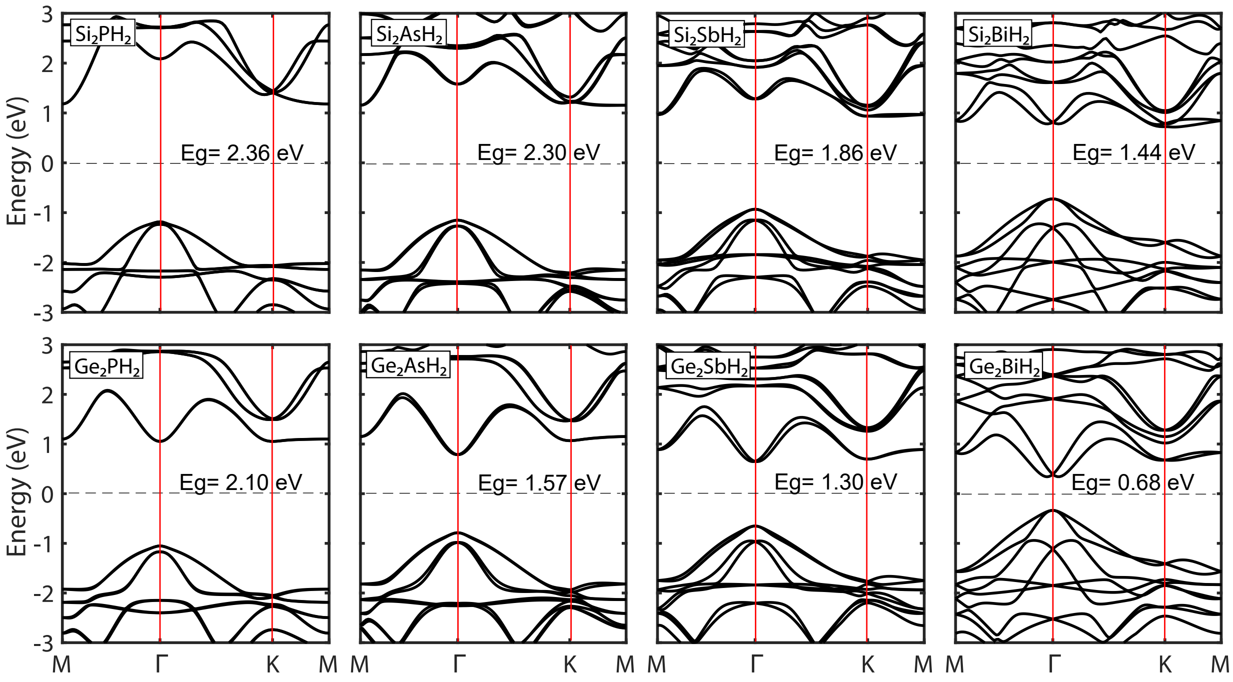

FIG. S2. Energy band structures of the X_2YH_2 binary compound monolayers along the main high symmetry k-points in the presence of spin-orbit coupling (SOC) together with the band gap amounts. The Fermi level has been set to zero.

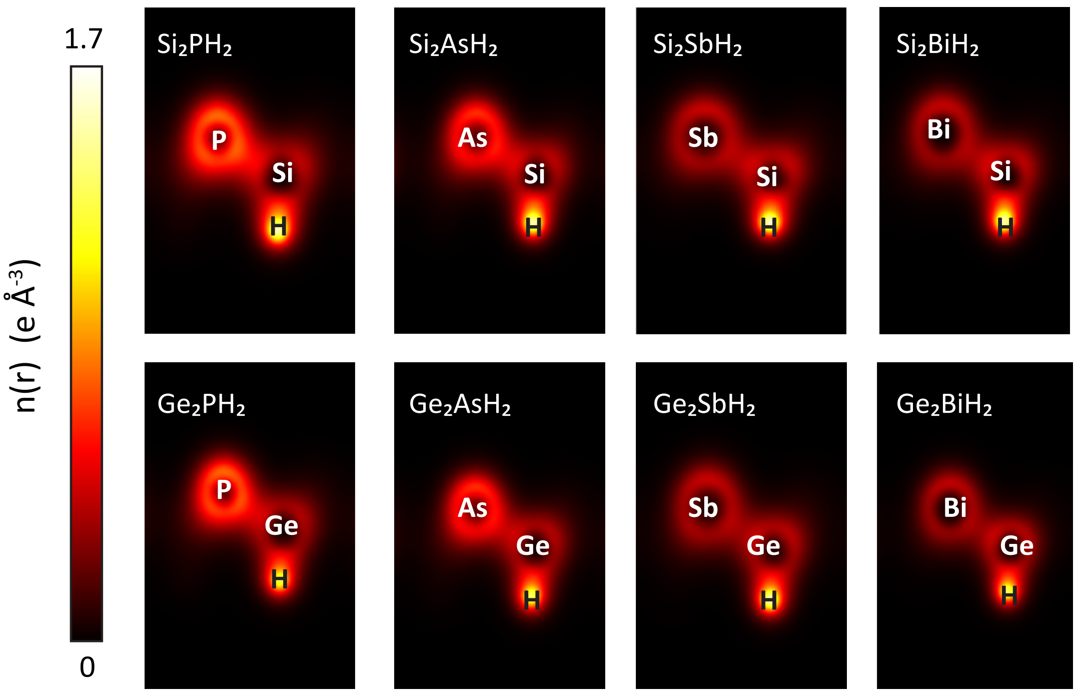

FIG. S3. Electron density of the X_2YH_2 binary compound monolayers at GGA level.

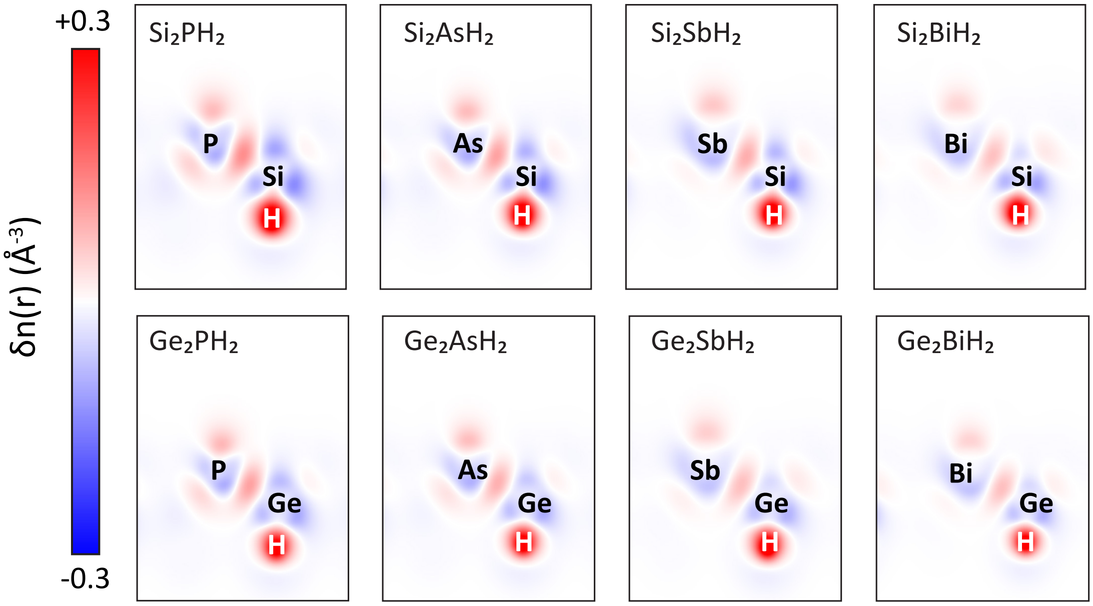

FIG. S4. Electron difference density of the X_2YH_2 binary compound monolayers at GGA level.

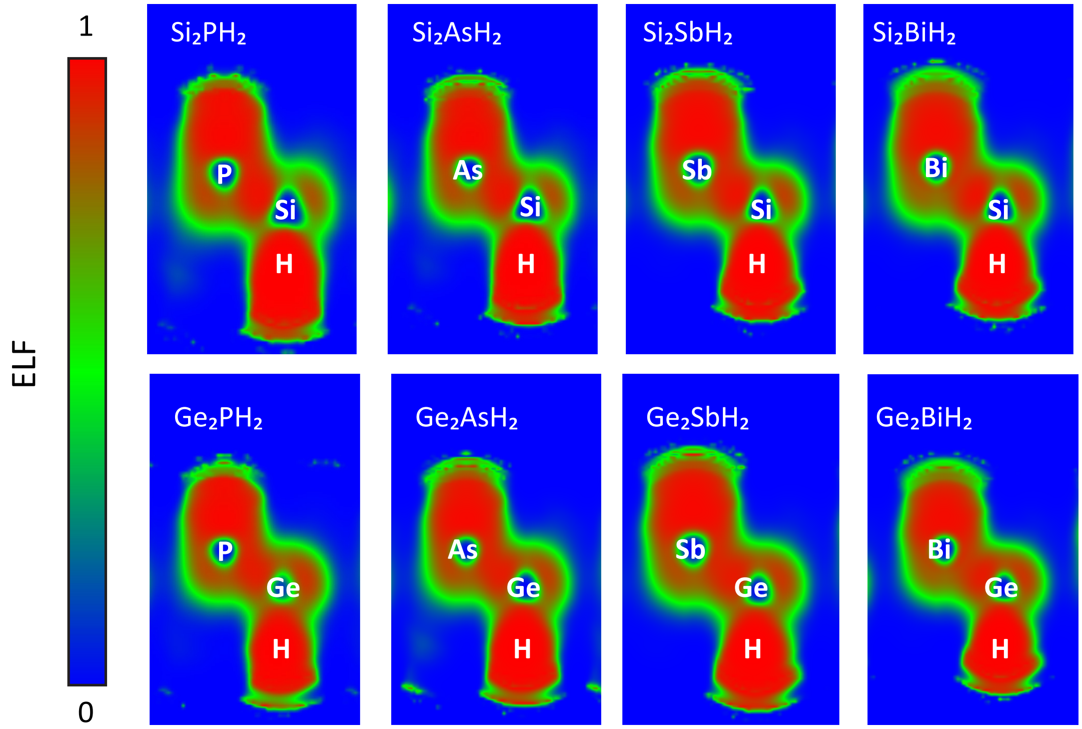

FIG. S5. The electron localization function (ELF) of the X_2YH_2 binary compound monolayers at GGA level.
